# Effects of manganese on routine semen quality parameters: results from a population-based study in China

**DOI:** 10.1186/1471-2458-12-919

**Published:** 2012-10-29

**Authors:** Yuyan Li, Junqing Wu, Weijin Zhou, Ersheng Gao

**Affiliations:** 1 Shanghai Institute of Planned Parenthood Research, Shanghai/WHO Collaborating Center on Human Research on Reproductive Health, 2140 Xietu Road, Shanghai, 200032, China; 2 School of Public Health, Fudan University, Shanghai, 200032, China; 3 National Laboratory of Contraceptives and Devices Research, Shanghai, 200032, China

## Abstract

**Background:**

Manganese (Mn) is an essential element in humans but its effect on semen quality is unclear. This study therefore aimed to assess the effects of Mn on semen quality in healthy men with no occupational exposure to Mn.

**Methods:**

Semen samples were obtained from healthy Chinese men 20–59 years old who were recruited from six provinces in China. Individuals with urogenital tract diseases, tuberculosis, or occupational exposure to heavy metals were excluded. A questionnaire survey was conducted, and the external genitalia, semen quality, and serum Mn levels were examined.

**Results:**

A total of 1,179 volunteers were enrolled in this study. The median serum Mn concentration was 8.2 μg/L (25th percentile (P_25_)=3.7 μg/L, P_75_=16.2μg/L). After adjusted area (six provinces), abstinence interval, season, registered residence, age of subjects, education level, income, smoking, and drinking, the risk of teratospermia was increased at serum Mn concentrations >19.40 μg/L (P_80_) group, with an adjusted odds ratio of 2.27 (95% confidence interval: 1.18–4.37).

**Conclusion:**

High serum Mn levels appeared to have harmful effects on sperm morphology and motility among healthy men with no occupational exposure to Mn.

## Background

Manganese (Mn) is an essential element for humans in small quantities, but a potent toxin at high levels. It is one of the most abundant elements and is widely distributed in soil, air, water, and food [1]. It is required for ubiquitous enzymatic reactions. Mn has a unique redox chemistry compared to other d-block elements, with several accessible oxidizing states [2]. High intracellular Mn levels protect against oxidative damage in various organisms, and oxidative protection by Mn is provided by the Nramp transporters [3]. One study clarified that the structural flexibility caused by Mn^2+^ is also important for enzyme dynamics, because Mn is required for RNA polymerization [4]. Mn^2+^ has been shown to be a chain-breaking antioxidant in biological systems. Mn activates several polymerases at low concentrations, but inhibits them at higher concentrations.

Studies [5-7] have shown that trace elements such as Mn and zinc (Zn) can alter reproductive functions, with effects on semen quality and infertility. The absence of essential heavy metals like Zn, Mn, may lead to the inhibition of enzyme systems required for sperm motility.Seminal Zn concentration was correlated with sperm count and the duration of abstinence in subfertile men. High seminal Zn levels may have an adverse effect on the spermatozoa-zona pellucida-induced acrosome reaction in normozoospermic men [8]. Another study showed that Mn at a concentration of 0.1 mM also supported good sperm motility (53%) in bovine semen at 6 h *in vitro*, compared to the control without Mn^2+^ (28%) [9]. Moreover, 0.1 mM MnCl_2_ had beneficial effects on the maintenance of sperm motility with no detrimental effects on mucus penetration and fertilizing ability. Mn^2+^ is a potent stimulator of sperm motility through the stimulation of adenylate cyclase activity [9].

In contrast, the results of animal experiments and studies of human occupational Mn exposure have indicated that exposure to high levels of Mn might impair male fertility. Elbetieha et al. postulated that ingestion of high doses of MnCl_2_ by male and female mice had adverse effects on fertility and reproduction [10]. MnSO_4_ adversely affected semen quality index and sperm viability in broiler breeder semen *in vitro* at concentrations of ≥6,500 mg/L [11].

Few studies have examined the effects of Mn exposure on male reproductive health [9]. Although some studies [4,6,10,11] have focused on the harmful effects of Mn on semen parameters in animals and occupationally-exposed men, there was a study to show that the Mn had no harmful to semen quality [12]. Moreover, the effects of Mn on humans without occupational exposure remain unclear. There is currently no information on threshold values or normal ranges for the physiological, rather than toxic effects, of Mn on semen quality or other parameters. This study therefore investigated the possible impacts of Mn on semen quality among healthy men with no occupational exposure to Mn, to determine its effects on semen quality and male reproductive health.

## Methods

### Study field and population

This study was conducted in a total of six cities from Hebei, Henan, Shanxi, Guizhou, Zhejiang and Shandong provinces between January, 2001 and December, 2004. According to the calculated formula for sample size, a minimum of 983 samples were required. Two hundred subjects were enrolled from each province using a two-stage sampling strategy. Fifteen counties were selected during the first stage of sampling, and volunteers from the 15 participating counties were recruited during the second stage. Adult males, 20–59 years old, who had lived in the respective cities more than 10 years, and who had no reproductive disorders, or no identifiable history of infertility, were enrolled. Individuals with chronic diseases such as diabetes, kidney disease, atherosclerosis, vascular disease, genital diseases, or hypertension, or worked in factory related to metal machining, were excluded from the study. When recruiting the subjects, IEC about the contents and criterion of subjects were conducted, so as to make enough males know this study and willing to participate in. A small gift was given to each subject, after the questionnaire and physical examination finished.

The study was conducted with the approval of the ethical committee of Shanghai Institute of Planned Parenthood Institute Research. A total of 1,179 volunteers who met the inclusion criteria were finally recruited. The subjects were informed about the study by trained staff, and informed consent was obtained. The participants each completed a questionnaire survey, underwent a physical examination, and their semen quality and levels of trace elements were analyzed.

### Questionnaire survey and physical examination

All qualified subjects completed a face-to-face interview questionnaire, which included information about demographic parameters, living habits, and diseases of reproductive organs (previous or current genital diseases such as cryptorchidism, inguinal hernia, varicocele, epididymitis, gonorrhea, chlamydia, and surgery for torsion of the testis) (the main contents of questionnaire were listed in the Additional file 1: Questionnaire survey and record). Physical examinations were performed by a trained physician at each site. A general and reproductive health examination was conducted, including scrotal palpation and Prader orchidometer measurement to assess testicular volume. The location and size of each testis was recorded and the possible presence of varicocele and hydrocele were checked.

### Semen parameters measurement

Semen specimens were collected after a period of 3–7 days of sexual abstinence. The duration of sexual abstinence and season of semen collection were recorded. Semen specimens were collected at the local family planning institution by masturbation, and delivered immediately to the laboratory in the same building. The room temperature during masturbation was kept between 18°C and 25°C.

All the semen samples were kept at 37°C to liquefy for routine semen analysis, according to World Health Organization (WHO) guidelines [13]. The appearance of the semen sample, semen volume and viscosity, sperm density, liquefaction time, total sperm count, percentage of motile spermatozoa (percentage of sperm with rapid and linear progressive motility and sluggish linear motility), and percentage of normal sperm forms were examined by trained laboratory technicians.

Semen volume was evaluated on the basis of semen weight, assuming a density of 1.0 g/mL. The plastic container was weighed before and after sample collection, and the difference between the weights was recorded as the volume. The sperm motility percentage was measured using the known volume of the specimen, which was placed onto a clean glass slide and covered by a coverslip, and examined under positive phase-contrast microscopy at a magnification of ×400. The proportion of progressively motile sperm in the four motility categories (fast progressive sperm, slow progressive sperm, non-progressive sperm and immotile sperm) was assessed, with the help of an ocular grid. A 1-mL sample was diluted (1: 20) with formaldehyde to examine the sperm concentration using an improved Neubauer hemocytometer. Sperm were counted in four to six randomly chosen fields. For morphological classification, 100 spermatozoa were counted using a high-quality phase-contrast microscope (magnification ×600). Thin, well-spread smears were air-dried, fixed, and stained according to the Papanicolaou method. The classification, including head shape/size defects, neck and midpiece defects, tail defects, and cytoplasmic droplets, was based on the manual published by WHO [13].

Quality control methods included using the same reagents, same test methods, and same standards in all six study centers. In addition, three training workshops on the examination of semen quality were held as part of the study. Semen parameter examination procedures were performed twice, and if the disparity between the results was >10%, a third measurement was made by a third laboratory technician, and the mean of the closer observations was used.

### Serum Mn examination

After the blood samples were collected in plastic centrifuge tube, serums were extracted, and they were rapidly placed at below −70°C refrigerators for storage. The samples from each center were transported to the National Minerals Examination Center in Shanxi province for examination. Serum Mn concentrations were measured by atomic absorption spectrophotometry in the National Laboratory of the Ministry of Geology and Mineral Resource, by specialized personnel. Atomic absorption spectroscopy is a well-established technique for the determination of trace elements, and is generally more sensitive and less subject to interference than either arc spectroscopy or flame photometry [14].

Examination was performed using an Excell Inductively Coupled Plasma Mass Spectrometer (TJA) with the following settings: power, 1,350 W; plasma gas: 15 L/min; carrier gas: 1 L/min; aux gas: 0.6 L/min; volume of sample extracting: 1.5 mL/min. First, 0.5 mL serum sample was mixed with 3.5 mL 1% HNO_3_ and 1 mL H_2_O_2_ for 12 h and dried by distillation. HNO_3_ 1 mL was added and the sample was dried, followed by the addition of 0.5 mL HNO_3_. Finally 10 mL of the sample was used for detection. A parallel sample and a standard sample were examined at 10-sample intervals for quality control. The proportions of Mn recovered from the standard sample were 86.5%, 94.3%, 109.2%, 68.7%, and 94.7%, respectively.

### Statistical analysis

All data were managed and analyzed by the Shanghai Institute of Planned Parenthood Research. Coding, double data inputting were conducted independently by two people using Epi-info software. And the database was checked whether the datum were consistent with the questionnaire survey results. Data were analyzed using SAS 9.1 software. The rank sum test, Spearman’s rank correlation, linear regression, ANOVA, χ^2^ and logistic regression analyses were used in this study.

Serum Mn concentrations were compared among subjects from the six provinces, and between men with normal and abnormal semen qualities, using rank sum tests. Spearman’s rank correlation and linear regression were used when Mn was measured as a continuous variable. The Mn level was divided into five groups by the 20th, 40th, 60th, and 80th percentiles, with cut-off points of 2.8 μg/L, 6.4 μg/L, 10.7 μg/L and 19.4 μg/L, and differences in semen parameters were analyzed by ANOVA. Semen parameters were also divided into two groups according to the reference value recommended by WHO [13] and analyzed by χ^2^ tests to explore variations in abnormal proportions of sperm among the five Mn groups. Finally, the categorized semen parameter variables were taken as dependent variables for logistic regression, with Mn as an independent variable and the 40–60% concentration group as a reference group, because Mn is a necessary trace element for humans [1-3]. Although some studies have found beneficial or harmful effects of Mn in animals and humans, including in terms of semen quality [3,4,9-11], the threshold values and normal ranges for the physiological, rather than toxic effects of Mn on semen quality and other parameters remain unknown. We therefore assumed that Mn concentrations around the median value had no significant physiological effects, and the 40–59% Mn concentration group was therefore used as the reference group for logistic regression. Other factors potentially influencing semen quality, including area, season of semen sample collection, abstinence interval, registered residence (rural or urban), age, education, income, and smoking and drinking habits were adjusted as confounders in multi-logistics models.

## Results

### General characteristics

A total of 1,179 eligible subjects were recruited from six cities, including 196 from Hebei, 184 from Henan, 199 from Shanxi, 193 from Guizhou, 205 from Zhejiang and 202 from Shandong, respectively.

The average age was 37.1±9.9 years. Subjects from 20–29 years accounted for 29.3%, and those aged 30–39 years for 29.0%. Most of men had graduated from junior high school (39.2%) and senior high school or alternative (32.5%), but 8.9% had education only to primary school or below, and 19.4% to college level or higher. The subjects’ occupations included peasant (31.3%), factory worker (21.1%), doctor/teacher/researcher/ professional (22.6%), commercial service providers (13.1%), and other employment (11.8%). The average income yearly was 5,000 Yuan (approximately 735 US dollars), and 31.30% of subjects’ income was in the 2,000–4,999 Yuan group (Table 1). The age, education level, occupation and income among six provinces were statistical different, significantly (P<0.05) (Table 1).

**Table 1:** Subject’s demographics among six provinces (%)

**Characteristics**	**Hebei**	**Henan**	**Shanxi**	**Guizhou**	**Zhejaing**	**Shandong**	**Total**
**Age**							
20–29	24.0	52.1	27.2	25.4	23.9	25.3	29.3
30–39	28.6	44.5	23.6	26.9	27.3	24.3	29.0
40–49	27.5	2.2	24.1	24.9	30.2	31.1	23.7
50–59	19.9	1.2	25.1	22.8	18.5	19.3	18.0
**Education**							
Primary school or lower	6.1	7.6	5.5	10.9	10.2	12.9	8.9
Junior high school	39.3	44.6	36.2	45.1	41.5	29.2	39.2
Senior high school or alternative	46.9	20.1	38.2	26.9	33.2	28.7	32.5
College or higher	7.4	27.7	20.1	17.1	15.1	29.2	19.4
**Occupation**							
Peasant	28.1	40.3	42.7	27.5	20.5	29.9	31.2
Factory worker	34.7	8.8	27.1	20.2	17.1	17.9	21.0
Commercial service worker	4.6	19.3	8.5	17.6	17.1	11.9	13.1
Doctor/teacher/researcher	26.0	23.9	14.1	14.5	34.6	22.4	22.6
Other	6.6	7.7	7.5	20.2	10.7	17.9	12.1
**Income level (Yuan)**							
<2,000	28.1	33.2	17.6	16.6	0.5	4.0	16.3
2,000–4,999	59.7	27.7	39.2	26.9	5.4	29.7	31.3
5,000–9,999	11.7	16.8	38.2	39.4	18.0	24.7	24.9
10,000-	0.5	22.3	5.0	17.1	76.1	41.6	27.6
**Registered residence**							
Urban	57.7	46.7	50.3	50.8	56.1	57.4	53.3
Rural	42.3	53.3	49.8	49.2	43.9	42.6	46.7

A total of 53.3% of men were from urban areas, which was slightly higher than the percentage from rural areas (46.7%). The distribution of registered residence had no statistical difference among six provinces (p=0.160) (Table 1).

### Analysis of semen parameters

Subjects were recruited, and semen samples were collected, in different seasons: 30.4% in spring, 21.0% in summer, 24.9% in autumn, and 23.8 % in winter. The abstinence intervals prior to semen collection were 3–7 days.

According to the Laboratory Manual for the Examination of Human Semen and Sperm–Cervical Mucus Interaction, third edition (WHO) guidelines [13], the proportion of samples with lower than normal semen volume (<2 mL) was 22.1% (mean: 2.5±1.1 mL). The Mean sperm density was 72.2±42.6×10^6^/mL, and the proportion with of oligospermia (sperm density <20×10^6^/mL) was 4.8%. The mean values for sperm viability, progressive motility, and normal morphology were 69.9±13.0%, 43.4±15.8%, and 42.3±13.1%. The proportions with sperm viability <75%, asthenospermia (progressive motility <50%) and teratospermia (normal morphology <30%) were 61.9%, 58.3% and 12.9%, respectively.

The five semen routine parameters were statistical significant difference among six provinces (Table 2).

**Table 2:** Comparison of semen parameters among six provinces (ANOVA)

	**N**	**Semen volume (ml)**	**Sperm density (×10**^**6**^**/ml)**	**Sperm viability (%)**	**Progressive motility (%)**	Normal morphology (%)*
Hebei	196	2.4 (0.4) ^b^	63.5 (23.3) ^b^	66.6 (5.0) ^c^	42.1 (4.9) ^c^	33.3 (1.8) ^e^
Henan	184	2.2 (1.2) ^c^	84.0 (50.5) ^a^	68.6 (16.4) ^c^	46.9 (14.1) ^b^	54.9 (21.9) ^b^
Shanxi	199	2.1 (0.3) ^c^	85.9 (36.3) ^a^	58.9 (11.2) ^d^	53.2 (10.8) ^a^	44.8 (11.5) ^c^
Guizhou	193	2.4 (1.2) ^b^	53.2 (33.9) ^c^	78.4 (8.2) ^a^	43.7 (9.5) ^c^	37.7 (8.04) ^d^
Zhejiang	205	2.6 (1.5) ^b^	80.3 (57.8) ^a^	74.3 (15.5) ^b^	48.2 (18.7) ^b^	33.8 (12.9) ^e^
Shandong	202	2.9 (1.0)^a^	66.2 (33.9) ^b^	72.3 (7.8) ^b^	49.0 (12.9) ^b^	59.4 (12.8) ^a^

Note: Values given as mean ±standard deviation. *P<0.05. ^*a, ,b*^ denote different means if the letters are not the same.

### Compare of Mn concentration

The Mn concentration was <50 μg/L in most cases, with a median of 8.2 μg/L (P_25_=3.7 μg/L; P_75_=16.2 μg/L). The serum Mn concentrations differed among the six provinces (P<0.0001), with the median concentrations in Hebei and Henan provinces being the highest: 12.8 μg/L (P_25_–P_75_: 7.0–22.0) and 11.5 μg/L (P_25_–P_75_: 6.5–22.0), respectively. The serum Mn levels in Guizhou and Shanxi were 9.8 μg/L (P_25_–P_75_: 5.2–13.8) and 8.0 μg/L (P_25_–P_75_: 4.4–14.7), respectively, while they were lowest in Zhejiang (5.3 μg/L, P_25_–P_75_: 2.0–9.8) and Shandong (3.9 μg/L, P_25_–P_75_: 0.4–10.8).

Semen parameters can be divided into two groups (normal and abnormal), according to the WHO-recommended reference values [13], and the Mn levels were compared between these two groups using rank sum tests. There were significant differences in Mn concentrations between men with normal and abnormal of sperm viabilities and progressive motilities (Table 3). The median serum Mn level in the group with abnormal sperm viability was 9.0 μg/L, compared to 7.7 μg/L in the group with normal viability. The median serum Mn level in the group with abnormal progressive motility was 9.1 μg/L, which was higher than in the group with normal progressive motility (7.4 μg/L).

**Table 3:** Comparison of serum Mn contents according to semen quality groups (rank sum tests)

**Semen parameter**	**Group**	**N**	**Median**	**25th–75th percentile**	**P value**
Semen volume	normal	919	8.5	3.7–17.3	0.413
	abnormal	260	7.8	3.9–13.8	
Sperm density	normal	1123	8.1	3.6–16.2	0.300
	abnormal	56	10. 5	4.5–19.3	
Sperm viability	normal	449	7.7	2.9–15.3	**0.031**
	abnormal	730	9.0	4.0–16.6	
Progressive motility	normal	492	7.4	2.9–14.3	**0.0003**
	abnormal	687	9.1	4.4–17.7	
Normal morphology	normal	1026	8.1	3.7–15.9	0.283
	abnormal	153	7.7	2.8–15.9	

Notes: abnormal values were semen volume <2 mL; sperm density <20×10^6^/mL; sperm viability <75%; progressive motility <50%; normal morphology <30%.

### Possible impacts of Mn on semen parameters

Spearman’s rank correlation analysis showed that the serum Mn level was weakly negatively correlated with progressive motility (r=−0.082) and normal morphology (r=−0.072) (P<0.05). The linear regression model found that Mn had a negative impact on progressive motility, with a parameter estimate for the linear regression model of −0.029 (P=0.021). Multi-linear regression, however, failed to show a significant effect of Mn as a continuous variable on semen parameters, even after adjusting for area (6 groups), abstinence interval (3–7 days, ranked variable), seasons (spring, summer, autumn, winter), registered residence (rural and urban), age group (20–29; 20–29; 40–49; 50–59), educational level (primary school or lower, junior high school, senior high school or alternative, college or higher) , income (<2,000 Yuan, 2,000–4,999Yuan, 5,000–9,999 Yuan, ≥10,000 Yuan), smoking (yes or no) and drinking (yes or no) (P>0.05).

Univariate ANOVA showed that the percentage with normal morphology was significantly different between the five Mn groups. The percentage showing normal morphology was highest in men with Mn levels <2.8 μg/L (P_20_) (46.2±17.6%), compared to 41.9–43.0% in the other groups (Table 4).

**Table 4:** Comparison of semen parameters according to percentile of Mn concentration (ANOVA)

**Mn group**	**N**	**Semen volume (ml)**	**Sperm density (×10**^**6**^**/ml)**	**Sperm viability (%)**	**Progressive motility (%)**	Normal morphology (%)*
<20%	234	2.6 (1.1)	70.9 (51.9)	71.4 (12.5)	49.1 (13.9)	46.2 (17.6)^a^
20–39%	232	2.5 (1.1)	69.9 (37.4)	69.2 (13.6)	47.0 (14.2)	41.9 (13.6)^b^
40–59%	241	2.4 (1.1)	77.0 (44.6)	69.3 (14.8)	47.6 (13.9)	43.0 (15.4)^a,b^
60–79%	236	2.4 (1.0)	73.1 (40.0)	69.8 (12.2)	47.0 (11.8)	43.0 (15.2)^a,b^
≥80%	236	2.5 (1.0)	69.9 (37.3)	69.9 (11.6)	45.7 (11.4)	42.6 (16.7)^a,b^

Note: Values given as Mean(Sd). *P<0.05. ^*a, b*^ denote different means if the letters are not the same.

The differences in proportions of asthenospermia among the five Mn groups are shown in Table 5. Abnormal progressive motility increased with increasing Mn levels (P=0.0004, Cochran-Mantel-Haenszel χ^2^ test). The proportion with abnormal sperm viability differed slightly among the five Mn groups, and the P value was close to 0.05 (P=0.054), with the lowest proportion of abnormal sperm viability in men below the 20th percentile for Mn concentrations.

**Table 5:** Proportions of abnormal sperm parameters according to Mn group (%)

**Mn group**	**N**	**Semen volume**	**Sperm density**	**Sperm viability**	**Progressive motility**	**Normal morphology**
<20%	234	22.3	3.0	54.5	48.9	15.2
20–39%	232	20.3	5.1	64.7	59.5	15.0
40–59%	241	27.3	4.6	59.5	55.8	11.7
60–79%	236	23.4	6.0	65.5	60.9	8.6
≥80%	236	16.9	5.1	65.4	66.2	14.2

The effects of Mn concentrations on semen quality were examined by logistic regression analyses, using the 40–59% Mn concentration group as the reference group (Table 6). Univariate logistic regression found that the ≥80% group had a lower risk of semen volume decline (odds ratio (OR)=0.54, 95% confidence interval (CI): 0.35–0.84), but a higher risk of asthenospermia (OR=1.56, 95%CI: 1.07–2.25). After adjusting for area, abstinence interval, season, registered residence, age, education level, income, smoking, and drinking, multi-logistic regression showed that the 20–39% and ≥80% Mn groups had lower risks of semen volume decline, with ORs of 0.62 (95%CI: 0.39–0.98) and 0.55 (95%CI: 0.34–0.89), respectively. However, the risk of teratospermia was higher in the >80% group compared to the 40–59% group (OR=2.27, 95%CI: 1.18–4.37).

**Table 6:** Logistic analysis of effect of Mn concentration on semen quality

**Mn Group**	**N**	**OR**	**95%CI**		**aOR**	**95%CI**	
			**lower**	**upper**		**lower**	**upper**
Abnormal semen volume							
<20%	233	0.77	0.50	1.16	0.90	0.56	1.46
20–39%	232	0.68	0.44	1.04	**0.62**	**0.39**	**0.98**
40–59%	242	1.00	—	—	1.00	—	—
60–79%	235	0.82	0.54	1.23	0.81	0.52	1.27
80%	237	**0.54**	**0.35**	**0.84**	**0.55**	**0.34**	**0.89**
Oligospermia							
<20%	233	0.65	0.25	1.71	0.43	0.16	1.19
20–39%	232	1.15	0.50	2.65	1.01	0.46	2.63
40–59%	242	1.00	—	—	1.00	—	—
60–79%	235	1.33	0.59	2.99	1.83	0.77	4.32
80%	237	1.12	0.48	2.59	1.49	0.62	3.61
Abnormal sperm viability							
<20%	233	0.82	0.57	1.17	0.88	0.56	1.41
20–39%	232	1.25	0.86	1.81	1.49	0.94	2.35
40–59%	242	1.00	—	—	1.00	—	—
60–79%	235	1.29	0.89	1.88	1.15	0.72	1.84
80%	237	1.29	0.89	1.86	1.06	0.66	1.69
Asthenospermia							
<20%	233	0.76	0.53	1.09	0.91	0.58	1.42
20–39%	232	1.16	0.81	1.68	1.34	0.87	2.06
40–59%	242	1.00	—	—	1.00	—	—
60–79%	235	1.23	0.86	1.77	0.93	0.59	1.46
80%	237	**1.56**	**1.07**	**2.25**	1.33	0.85	2.10
Teratospermia							
<20%	230	1.35	0.79	2.31	0.98	0.52	1.89
20–39%	220	1.33	0.77	2.29	1.01	0.54	1.88
40–59%	230	1.00	—	—	1.00	—	—
60–79%	221	0.71	0.38	1.31	0.92	0.46	1.84
80%	219	1.24	0.71	2.16	**2.27**	**1.18**	**4CP37**

Note: aOR means the odds ratio adjusted for area, abstinence interval, season, registered residence, age, educational level, income, smoking and drinking.

## Discussion

Since the 1990s, reports about decreasing human sperm counts and increasing abnormalities in human testes have focused research on the possible impacts of environmental factors such as lifestyle, environmental and occupational exposure to trace elements, environmental pollution, and pesticides on semen quality and male reproductive health [15,16]. Merzenich et al. [17] suggested that some meta-analyses of sperm-count data showed a global downward trend, but this conclusion should be interpreted with caution because of the heterogeneities among the studies in terms of geographical and/or ethnic variations, study designs and/or different methodological standards. More population-based studies are also needed to investigate the trends in male reproductive disorders and to explore the factors influencing male reproductive health.

Mn is a necessary element for humans, but although it has many important functions for normal reproductive health, overexposure may be toxic to reproductive health [7-9]. One study reported that high Mn levels were associated with erectile dysfunction, but this study only involved workers with severe sickness induced by occupational exposure to Mn [18]. The damaging effects of Mn on male reproductive function have mainly been examined in occupationally-exposed men, and showed prolonged time to semen liquefaction and decreased sperm motility among workers in contact with Mn, and decreasing percentage of motile sperm with increasing duration of employment as a miner [19]. In workers who had been in contact with Mn for more than 10 years, the activities of protective enzymes such as catalase in erythrocytes were lower than in controls, suggesting that high Mn levels might result in decreases in the activities of antioxidant enzymes, resulting in subsequent damage [20].

The role of Mn^2+^ was examined during the cryopreservation of cattle bull semen, and the addition of Mn to the egg-yolk-citrate extender + glycerol (EYC-G) dilutor was found to improve the quality/fertility of the semen, resulting in improved success rates of *in vitro* fertilization and artificial insemination [21]. An *in vitro* experiment using semen with >75% motility and 80×10^6^ sperm/mL from healthy males found that Mn^2+^ supplementation improved total thiols and reduced glutathione levels under normal and oxidative stress conditions, and that Mn^2+^ supplementation maintained the thiol level by reducing oxidative stress [22]. *In vitro* experiments using semen samples obtained from 12 normozoospermic healthy volunteer donors aged 19–23 years found that 50 μmol/L Mn(III)TMPyP, a kind of superoxide scavengers, named Mn(III) tetrakis (1-methyl-4-pyridyl) porphyrin, could attenuate the effects of superoxide (O2^+^) on sperm motility parameters [23]. In contrast, another experiment using semen from healthy male volunteers showed that 500 ppm Mn^2+^ significantly inhibited sperm motility with no accompanying change in seminal malondialdehyde levels, indicating that 500 ppm Mn^2+^ could affect sperm motility *in vitro*[24].

A potential role of Mn in infertile males has been reported. Lafond et al. [25] reported lower Mn levels in seminal plasma from men with lower sperm densities. Among 52 Nigerian male partners of infertile couples, normospermic infertile patients had higher serum Mn levels compared to those with oligospermia and azoospermia (P<0.001) [26], suggesting a potential role for Mn in the evaluation of infertile males. Another study, however, showed higher seminal Mn levels in infertile men compared to controls [27]. In a study of 200 infertile men, high Mn levels were associated with increased risk of low sperm motility (OR=5.4; 95%CI: 1.6–17.6) and low sperm concentration (OR=2.4; 95%CI: 1.2–4.9) [12]. There are thus conflicting results regarding the effects of Mn on semen quality in infertile men, and more studies are needed to confirm its impacts and to explore the dose threshold values for infertility.

Few studies have investigated the harmful impacts of Mn on reproductive function in men without occupational Mn exposure [16,22]. Some studies examined the *in vitro* effects of Mn on semen quality in healthy men [10,22,23] and found some useful results, but no consistent conclusions. The current study found that serum Mn concentrations >19.40 μg/L (P_80_) had negative impacts on sperm morphology, suggesting that high serum Mn levels can adversely affect semen quality in healthy men with no occupational Mn exposure, however, the value of 19.40 μg/L was only the 80th percentile cut-off point for all subjects, and could not be considered as the threshold value for the harmful effects of Mn on sperm morphology. No apparent risks were associated with lower (<20%) or higher (>80%) Mn levels in terms of semen volume, and a lower proportion of abnormal semen volume was found in the >80% Mn group, but semen quality assessment should not focus only on semen volume. In this study, the negative impacts of high Mn levels on sperm viability, progressive motility, and morphology were more obvious than the beneficial effects. Our results indicated that higher Mn concentration might be harmful to semen routine parameters among no-occupational Mn-contact men. So, long-term or lage amount of Mn contact should be avoid in life, especially for people at reproductive age.

However, this study was a cross-sectional survey and was therefore unable to demonstrate any cause-effect relationships. In addition, only serum Mn concentrations were measured, which do not fully reflect the status of Mn in the seminal plasma. Further studies are therefore needed to explore the effects of Mn on human semen quality and reproductive function.

## Conclusions

Mn is an important element in terms of semen quality and fertility, and is also an industrial material. Despite inconsistent results regarding the effects of Mn in infertile men, high levels of Mn appear to have negative impacts on semen quality and male reproductive health. It is therefore reasonable to advise men to avoid contact with high levels of Mn, especially in occupations related to Mn manufacture and the Mn industry.

## Competing interests

The authors declare that they have no competing interests.

## Authors’ contributions

All authors made substantial contributions to the conception and design of the paper. YL carried out the field questionnaire survey, performed the statistical analysis, and wrote the manuscript. JW participated in the study design and coordination. WZ coordinated the study and helped with data analysis. EG participated in the design of the study and helped to draft the manuscript. All authors read and approved the final manuscript.

## Pre-publication history

The pre-publication history for this paper can be accessed here:

http://www.biomedcentral.com/1471-2458/12/919/prepub

## Supplementary Material

Additional file 1 Questionnaire survey and record. Click here for file
