# Supplementary material for: Effects of manganese on routine semen quality parameters: results from a population-based study in China
Source: BMC Public Health. 2012 Oct 29;12:919. doi: 10.1186/1471-2458-12-919 (PMC3505748; doi:10.1186/1471-2458-12-919)
Supplement: Additional file 1 — Questionnaire survey and record. [file 1471-2458-12-919-S1.pdf]

Questionnaire survey and record

Id: □□□□□

Province _____________
County ____________
Name __________
Address___________________________
Phone number ____________

Excluded form

(a) How old are you? __________ (If age<20 or >60, then excluded)

(b) Do you have some reproductive dysfunctions or diseases (genital diseases such as cryptorchidism, inguinal hernia, varicocele, epididymitis, gonorrhea, chlamydia, and surgery for torsion of the testis) ?

1. Yes **(excluded)** 2. No

(c) Do you have some chronic disease, such as diabetes, kidney disease, atherosclerosis, vascular disease, genital diseases, or hypertension?

1. Yes **(excluded)** 2. No

(d) Do you work in factory related to Mn machining?

1. Yes **(excluded)** 2. No

(e) Do you word in or live near factories related to metal machining, such as lead, cadmium, zinc, copper, etc.?

1. Yes **(excluded)** 2. No

A. Demography characteristics ☐ for coding

A1 Birth date：______________ ☐☐☐☐☐☐

A2 Height ：_______cm ☐☐☐

A3 Weight ：_______kg ☐☐

A4 Education level：

1. Illiteracy 2. Primary school 3.Junior high school

4. Senior high school 5.junior college or higher ☐

A5 Registered residence：1.Urban 2.Rural ☐

A6 Occupation：

1. Peasant 2. Factory worker 3. Commercial service worker

4. Doctor/teacher/researcher 5. Other____________ ☐

A7 Do you have sexual intercourse? 1. Yes 2. No (**skip to A9**) ☐

A8 Age of first sexual intercourse?________year old ☐☐

A9 Marriage status：

1. Unmarried (**skip to A11**) 2. Married ☐

A10 Age of married: ___________

A11 Do you have child? 1. Yes 2. No ☐

A12 Income：________Yuan /person/year ☐☐☐☐☐☐

B. Living status

B1 Birth place： 1. Urban 2.Rural ☐

B2 How long do you live here? ____________year ☐☐

B3 Did you ever have enuresis? 1. Yes 2. No ☐

B4 Did you ever have mumps? 1. Yes 2. No ☐

B5 Do you smoke? 1. Yes 2. No (**skip to B7**) ☐

B6 Mean number of units smoked per day? ________ ☐☐

B7 Do you drink alcohol? 1. Yes 2. No (**skip to B10**) ☐

B8 What kind of alcohol? 1. Beer 2. White wine 3. Red wine

4. Other________ ☐

B9 Mean number of alcohol per day? ________ ☐☐

B10 Do you drink coffee? 1. Yes 2. No ☐

B11 Do you drink tea? 1. Yes 2. No ☐

B12 Do you work at sitting position long time? 1. Yes 2. No ☐

B13 Do you contact with pesticide often? 1. Yes 2. No ☐

B14 Do you contact with chemical materials, such as benzene, nitrogen sulfide, often?

1. Yes 2. No ☐

B15 Do you use mobile phone every day?

1. Yes 2. No（**skip to B17**） ☐

B16 Where the mobile phone is carried?

1. Trouser pocket 2. Bag 3. Other place ☐

B17 Do you use computer often? 1. Yes 2. No（**finished**） ☐

B18 How long do you use computer per every day? _____hours ☐

Signature of interviewer:______________

Data of examination:_______________

Records of semen quality examination

C1. Data for first semen sample collection: __________ □□□□□□

C2. Abstinence interval (day): _______ □

C3. Examination time after ejaculation (minute): _______ □□

C4. Appearance: 1.Normal 2. Abnormal □

C5. Time for liquefaction (minute): □

1. ≤20 2. 21~30 3. 31~60 4. >60

C6. Stickiness (length of drawbench): 1. ≤2cm 2. >2cm □

C7.Agglutination: 1.No 2. Have □

C8.Volume (ml): ____ □

C9. pH: __________ □

C10. Motility (100 sperm)

(a) rapid and linear progressive motility __________ □□

(b) sluggish linear motility __________ □□

(c) no linear __________ □□

(d) no motile__________ □□

C11. Density ($\times 10^6$/ml) __________ □□

C12. Total sperm count ($\times 10^6$) __________ □□

C13. Sperm viability (%) __________ □□

C14. Normal sperm forms (%) __________ □□

Signature of examination person:______________

Data of examination:_________________
